# Effects of Peanut Butter Supplementation on Older Adults' Physical Function: A 6‐Month Randomised Controlled Trial

**DOI:** 10.1002/jcsm.70221

**Published:** 2026-02-03

**Authors:** Ilili Feyesa, Jeew Hettiarachchi, Robin M. Daly, Elena S. George, Ekavi N. Georgousopoulou, David Scott, Brenton J. Baguley, Gavin Abbott, Sze‐Yen Tan

**Affiliations:** ^1^ School of Exercise and Nutrition Sciences Deakin University Geelong Victoria Australia; ^2^ Institute for Physical Activity and Nutrition (IPAN), School of Exercise and Nutrition Sciences Deakin University Geelong Victoria Australia; ^3^ Discipline of Nutrition and Dietetics, Faculty of Health University of Canberra Canberra Australia; ^4^ School of Medicine Sydney University of Notre Dame Australia Sydney Australia; ^5^ School of Clinical Sciences at Monash Health Monash University Clayton Victoria Australia

**Keywords:** muscle mass, muscle strength, nuts, older adults, physical function

## Abstract

**Background:**

Nut consumption has been associated with a reduced risk of functional decline, but evidence from randomised controlled trials to support functional benefit is lacking. Therefore, this study aimed to investigate whether daily supplementation of peanut butter over 6 months, relative to usual care, can improve physical function in community‐dwelling older adults.

**Methods:**

One hundred and twenty older adults (aged ≥ 65 years) at risk of falls were randomly assigned to receive peanut butter (43 g/day, *n* = 60) or maintain usual care (control, *n* = 60) for 6 months. Outcomes assessed at baseline and 6 months included physical function (4‐m gait speed [primary outcome], standing balance test, four‐square step test [FSST], five times sit‐to‐stand [5STS] test time and muscle power, 30‐s sit‐to‐stand (30‐s STS) and timed up and go [TUG] tests), muscle strength (handgrip [HGS] and isometric knee extensor strength tests [KES]) and anthropometry/body composition (weight, body mass index [BMI], total lean and fat mass and appendicular lean mass). Linear regression models, adjusting for age, sex, baseline value of the dependent variable, BMI, physical activity and diet quality, estimated intention‐to‐treat intervention effects.

**Results:**

A total of 108 (90%) participants completed the study. At baseline, 70% were female, and the mean ± SD age and BMI were 76.1 ± 4.6 years and 27.5 ± 4.2 kg/m^2^, respectively. At 6 months, there were no significant treatment effects on the primary outcome of gait speed or other measures of physical function (*p* > 0.05), with the exception that 5STS time and muscle power improved significantly more in the peanut butter compared to control group (estimated treatment effect: time, −1.23 s [95% CI, −2.09, −0.37], *p* = 0.006; absolute power, 22.0 W [95% CI: 7.1 to 36.9], *p* = 0.004; relative power, 0.27 W/kg [95% CI: 0.10 to 0.45], *p* = 0.002). Changes in HGS, KES, weight, BMI, total fat mass, total lean mass or appendicular lean mass did not differ between groups. In the peanut butter group, among those who completed the follow‐up, the mean (SD) adherence was 86.0 (13.8) %.

**Conclusion:**

In community‐dwelling older adults at risk for falls, daily peanut butter consumption for 6 months improved 5STS time and muscle power based on 5STS, but not gait speed, muscle strength or body composition.

**Trial Registration:** Australian New Zealand Clinical Trials: ACTRN12622001291774

## 1 Introduction

The ageing process is characterised by adverse alterations in skeletal muscle structure and function, leading to a progressive decline in muscle mass, strength and physical function [1]. Muscle mass and strength decline from around the fourth decade, progressively accelerating after 50 years of age, with lifetime losses of ~20%–30% in muscle mass and 40%–50% in muscle strength [2]. Physical function declines more rapidly after 65 years, declining by 50% or more from maximal capacity [2]. Collectively, these muscle‐related changes in older adults are associated with a range of adverse outcomes, including increased risk of falls and fractures [3], poor quality of life [4], loss of independence and hospitalisation [5]. Therefore, strategies to prevent or mitigate age‐related muscle and functional declines are a public health priority.

Good nutrition and resistance training are widely recognised as key strategies to mitigate age‐related declines in body composition and functional capacity [6]. Adequate dietary protein intake supports skeletal muscle anabolism by providing amino acids that stimulate postprandial muscle protein synthesis [7]. Systematic reviews of observational studies have noted an association between higher dietary protein intake and improved muscle strength and physical function [8]. Furthermore, the evidence from nutritional epidemiology to date suggests a positive association between healthier dietary patterns, such as the Mediterranean diet that includes nuts, and muscle function, including physical performance and mobility decline in older adults [9].

Nuts are a rich source of plant‐based protein, with peanuts and pistachios particularly notable for their high free amino acid content. In addition to protein, nuts are also abundant in vitamins, minerals, fibre, unsaturated fats and phytochemicals, all of which have anti‐inflammatory and antioxidant properties [10]. Chronic inflammation, characterised by elevated levels of biomarkers such as C‐reactive protein (CRP), interleukin‐6 (IL‐6) and tumour necrosis factor‐alpha (TNF‐α), has been associated with reduced muscle mass, strength and physical performance in older adults [11]. Evidence from systematic reviews and meta‐analyses of cohort studies and randomised controlled trials (RCTs) indicates that regular nut consumption may modestly attenuate inflammation and oxidative stress [12]. These anti‐inflammatory and antioxidant properties may elucidate the consistent associations between frequent nut intake and a reduced risk of cardiovascular disease [13]. In addition, nut supplementation in a butter form is a simple, practical, age‐appropriate and inexpensive dietary strategy that can be easily adopted by older adults. Its soft texture also makes it particularly suitable for individuals experiencing dentition issues, a common concern with ageing [14].

To date, several observational studies have explored the relationship between nut consumption and physical function in older adults. Nut consumption has been associated with a lower risk of poor muscle strength [15], sarcopenia [16], mortality and limitation in basic daily living ability [17]. It has also been linked with fewer and less severe functional limitations [18], reduced risk of impaired mobility in men and impaired physical function in women [19]. However, no intervention trials have evaluated the impact of nut consumption on muscle mass, strength or physical function in older adults. Therefore, the aim of this 6‐month RCT was to evaluate the effects of daily peanut butter supplementation on physical function, muscle mass and strength in community‐dwelling older adults at risk for falls.

## 2 Methods

### 2.1 Study Design and Population

This 6‐month RCT recruited 120 community‐dwelling older adults (aged ≥ 65 years) at risk for falls, defined by achieving a score of ≥ 2 points using a simplified falls risk screening questionnaire adapted from our previously used algorithm based on risk factors for falls in this population. The falls risk screening tool comprised six items. Age was scored as 0 points for < 70 years, 1 point for 70–75 years and 2 points for > 75 years. One point was allocated for each of the following: (i) having experienced two or more slips or trips (without a fall) in the past 12 months; (ii) feeling unsteady on their feet when standing, walking or performing daily activities; (iii) feeling at risk of falling; (iv) having difficulty rising from a chair or toilet without using their arms; and (v) taking four or more different types of medication [20]. The detailed protocol for this study has been published [21]. Briefly, participants were excluded for the following reasons: currently taking oral nutrition supplements (ONS); undertaking structured resistance or exercise programmes more than once a week; reported health conditions affecting dietary intake and metabolism (e.g., gastrointestinal disease, musculoskeletal conditions or cancer) or reported severe osteoarthritis, severe lower back pain, osteoporotic fracture or recent knee/hip replacements that would prevent them from performing physical function tests. Participants were recruited through social media, local area news bulletins and flyers and pamphlets distributed in the community, including at retirement villages, in Melbourne, Victoria.

The trial was conducted at the Institute of Physical Activity and Nutrition, Deakin University, Burwood Campus, Melbourne, Australia, and conformed to CONSORT guidelines. The intervention was conducted between May 2023 and July 2024. The study was approved by the Deakin University Human Research Ethics Committee (2022‐279) and was performed in accordance with the ethical standards as laid down in the 1964 Declaration of Helsinki and its later amendments. All participants provided written informed consent. The study was registered with the Australian New Zealand Clinical Trials Registry under ACTRN12622001291774.

### 2.2 Blinding and Randomisation

This was a single‐blind RCT design, wherein the researchers responsible for collecting outcome measures and conducting statistical analysis were blinded to the participants' group allocation. Participants were randomised in a 1:1 ratio to either the peanut butter group or the usual care control group following the baseline visit. A stratified randomisation allocation was developed using the Research Electronic Data Capture (REDCap) secure web application by a biostatistician who was not involved in the study. Randomisation was stratified by age (≤ 80 or > 80 years), sex (male or female) and body mass index (BMI) (≤ 25 or > 25 kg/m^2^).

### 2.3 Intervention and Control Groups

Participants were supplied with 43 g/day of peanut butter for a 6‐month trial (at no cost). The 43‐g/day peanut butter used in our study, approximately equivalent to 1.5 servings of nuts, supplied ~250 kcal, 20 g of fat (with over 90% being unsaturated) and 10 g of protein. In contrast, participants in the control group were advised to maintain their usual dietary habits and to refrain from consuming nuts throughout the trial period. Participants in both groups were instructed to maintain their usual levels of physical activity and exercise throughout the study period.

### 2.4 Adherence

Participants in the intervention group were instructed to return any unopened peanut butter containers during their 3‐ and 6‐month visits. Consumption was determined by subtracting the number of containers returned from the total number provided. Adherence to the intervention was calculated as the proportion (%) derived from the total number consumed to the total number of days in the intervention. A sensitivity analysis adhering to the per‐protocol principle was conducted, only including participants with ≥ 80% peanut butter consumption adherence.

### 2.5 Questionnaires and Anthropometry

Participants completed self‐administered questionnaires that collected information about general demographics and health at baseline.

For anthropometric measures, participants were instructed to empty their pockets and remove heavy clothing and shoes. Height (m) was assessed without shoes to the nearest 0.1 cm. Weight (kg) was measured to the nearest 0.1 kg, and BMI was calculated from height and weight (kg/m^2^).

The present study focuses on the primary (gait speed) and key secondary outcomes related to physical function and body composition. Outcomes on cognitive function and telomere length will be reported in separate publications.

### 2.6 Physical Performance

#### 2.6.1 Gait Speed

Gait speed was measured using the 4‐m gait speed test. Participants were instructed to walk a 4‐m distance on a flat surface at their usual pace, and the test was repeated twice. The time taken to complete the test was recorded using timing gates (Swift Speedlink Performance Equipment systems). No verbal encouragement was given during the test. Maximal gait speed was computed as the 4‐m distance divided by the elapsed time (m/s).

#### 2.6.2 5STS Time and Muscle Power

The 5STS was conducted using standard procedures. Participants were asked to stand up from a chair (seat height: 46 cm) five times with their arms crossed over their chest, using their maximum range of motion, as quickly as possible [22]. The time (seconds) taken to finish the test (recorded by a stopwatch) was used to calculate the absolute and relative STS muscle power by the following validated equation [23].

$$\begin{array}{c} \text{Absolute}\;\mathrm{STS}\;\text{muscle power}\;\left(\text{watt}\;\left(W\right)\right)=\\ \left[\text{Body weight}\;\left(\mathrm{kg}\right)\times 0.9\times g\times \left(\text{height}\;\left(m\right)\times 0.5-\text{chair height}\;\left(m\right)\right)\right]\\ ÷\left(5\;\mathrm{STS}\;\text{time}\left(s\right)\times 0.1\right) \end{array}$$

where g represents the acceleration due to gravity (9.81 m/s^2^).

$$\begin{array}{c} \begin{array}{c} \text{Relative}\;\mathrm{STS}\;\text{muscle power}\;\left(W/\mathrm{kg}\right)=\text{Absolute}\;\mathrm{STS}\;\text{muscle power}\;\left(W\right)\\ ÷\text{Body weight}\;\left(\mathrm{kg}\right) \end{array} \end{array}$$

#### 2.6.3 30‐s Sit‐to‐Stand Performance

The 30‐s STS performance was assessed as the number of times a person was able to rise and sit from a chair (seat height: 46 cm) within 30 s with their arms crossed over their chest, using their maximum range of motion, as quickly as possible.

#### 2.6.4 Four‐Square Step Test (FSST)

For the FSST, participants were asked to step forwards, sideways and backwards as quickly as possible over two rods arranged in a cross formation to create four squares, moving first in a clockwise direction and then in the counterclockwise direction. Participants were given one practice trial prior to administering the test. The shortest recorded time to complete the task, measured to the nearest 0.1 s, was used for analysis.

#### 2.6.5 Timed Up and Go Test (TUG)

For the TUG, participants were timed as they stood up from a chair without armrests, walked 3 m to a cone, turned around, walked back and sat down again. The timer was started on the command ‘go’ and stopped once the participant had returned to the chair and placed their back against the backrest. The time taken to complete the trial was recorded.

### 2.7 Muscle Strength

Upper and lower body muscle strength were assessed using handgrip strength (HGS) and knee extensor strength (KES), respectively. HGS (in kilogrammes) was measured using a calibrated handheld dynamometer (Jamar Plus, Digital Hand Dynamometer). Three alternating attempts for each hand were given; the maximum from any attempt was used as the participant's maximum HGS. Isometric KES (in kilogrammes) was measured via a strap attached to the participant's lower leg that was fixed to a strain gauge attached to a metal bar at the rear of a chair. Two attempts were given, and the maximum force exerted was recorded.

### 2.8 Body Composition

Whole‐body scans were performed using dual‐energy X‐ray absorptiometry (DXA) (Lunar iDXA; GE HealthCare, Australia and New Zealand) to determine whole‐body fat mass, lean soft tissue mass (LSTM) and body fat percentage. Upper‐ and lower‐limb LSTM were summed to calculate appendicular lean mass (ALM). DXA quality assurance was performed daily to ensure scanner reliability.

### 2.9 Late‐Life Function and Disability Instrument (LLFDI)

The LLFDI evaluates the frequency and limitations associated with performing major life tasks and engaging in social activities within the community [24]. As outlined in the LLFDI Manual, the raw summary scores for frequency and limitation were converted into scaled scores ranging from 0 to 100, utilising a Rasch model. A score of 100 indicates greater frequency or fewer limitations, whereas a score of 0 signifies lesser frequency or greater limitations.

### 2.10 Dietary Assessment

Participants completed 3‐day food records (two weekdays and one weekend day) at baseline (pre‐intervention) and post‐intervention. Participants were asked to complete records on typical days, maintain usual eating patterns and provide detailed entries including food type, brand, preparation method (e.g., boiled, fired and baked) and food quantities, which were recorded using household measures (e.g., cup and teaspoon). Intervention participants recorded their habitual diet at baseline. Returned records were reviewed and validated by a qualified dietitian for completeness. Dietary intakes were analysed using the FoodWorks 10 Professional software, which utilises nutrition composition data from the AusFoods (AUSNUT 2011–2013 and the new Australian Food Composition Database) and AusBrands databases.

Diet quality using participant food records was assessed using the Healthy Eating Index (HEI)‐2020 [25]. The HEI‐2020 comprises nine components related to adequacy—‘total fruit, whole fruit, total vegetables, greens and beans, whole grains, dairy, total protein foods, seafood and plant proteins, and fatty acids’—and four components related to moderation—‘refined grains, sodium, added sugars, and saturated fat’. A maximum of 5 points was allocated to ‘total fruit’, ‘whole fruits’, ‘total vegetables’, ‘greens and beans’, ‘total protein foods’ and ‘seafood and plant proteins’; and a maximum of 10 points to ‘whole grains’, ‘dairy’, ‘fatty acids’, ‘refined grains’, ‘sodium’, ‘added sugars’ and ‘saturated fats’. The total score ranges from 0 to 100, with a higher score indicating a greater intake of adequacy components and a lower intake of moderation components [25]. HEI‐2020 components and Cup Equivalent/Ounce Equivalent were matched with FoodWorks food group components and serve equivalents using the US Dietary Guidelines 2020–2025 [26].

### 2.11 Nutritional Status

The nutritional status of the participants was assessed by a trained researcher using the Mini Nutrition Assessment (MNA), a validated nutrition assessment tool in older adults. Participants were categorised as at risk for malnutrition or malnourished (< 23.5) based on the total MNA score (range 0–30).

### 2.12 Habitual Physical Activity

Habitual physical activity was assessed at baseline and at 6 months using the validated self‐administered Physical Activity Scale for the Elderly (PASE) questionnaire [27]. PASE measures the frequency, duration and intensity of leisure, household and occupational activities over the past week [27]. Scores range from 0 to 793, with higher scores reflecting greater levels of habitual physical activity.

### 2.13 Perception of Intervention

Participants' perception towards the peanut butter intervention was assessed using a self‐administered questionnaire during the visits at 3 and 6 months. This includes ratings of participants' perception of the taste, texture, appearance, flavour and the ease of following the peanut butter intervention using a 9‐point Likert scale.

### 2.14 Risk of Sarcopenia

The risk of sarcopenia was identified using the SARC‐F screening tool that encompasses five self‐reported questions on strength (S), assistance with walking (A), rising from a chair (R), climbing stairs (C) and history of falls (F), and which is recommended for use in older adults by the international sarcopenia guidelines.

### 2.15 Adverse Events

Participants were instructed to discontinue peanut butter consumption if they experienced any adverse events and to notify the research team. All reported events were recorded by the study team.

### 2.16 Sample Size

The sample size for this study was based on a 2021 trial that aimed to improve walking speed in older adults using an oral nutritional supplement (ONS) with a nutrient composition similar to peanut butter [28]. This study reported an average improvement in walking speed by 0.15 m/s. Previous research has reported that a 0.05‐ to 0.12‐m/s change in walking speed is a clinically meaningful change [29]. Therefore, based on an expected net improvement of 0.15 m/s in walking speed in the peanut butter compared to control group, assuming a standard deviation of 0.2 m/s [30], allowing an acceptable difference of 0.15 m/s between groups, and accounting for an expected attrition rate of 15%, a sample size of 60 participants per group (yielding a total sample size of 120) would provide 80% power (*p* < 0.05, two‐tailed).

### 2.17 Statistical Analysis

The hierarchy of primary and secondary outcomes, including the designation of usual gait speed as the primary outcome, is consistent with the preregistered protocol. Since the publication of our protocol, via consultation with a statistician, we have strengthened our statistical analysis. Instead of the originally planned linear mixed model, we used a regression model that serves a similar function, and we have used more appropriate methods for handling missing data in the intention‐to‐treat analysis.

Normality of continuous variables was evaluated using boxplots and the Shapiro–Wilk test. Descriptive statistics are presented as mean ± standard deviation (SD) for continuous variables and as frequencies (percentages) for categorical variables. Analyses were conducted on an intention‐to‐treat basis according to the treatment group assigned at randomisation. Missing data were addressed using multiple imputations by chained equations, performed separately for each treatment group to generate 20 complete datasets. Multiple imputation methods used an iterative Markov chain Monte Carlo method (STATA ‘mi’ command).

Within‐group changes in outcomes (95% CI) were estimated using linear regression models with change scores as the dependent variable. We analysed treatment effects using linear regression models, with the follow‐up value as the dependent variable and the baseline value, BMI, PASE score and HEI‐2020 as a covariate. The same modelling approach was applied in a per‐protocol sensitivity analysis restricted to participants achieving ≥ 80% compliance; results are presented in the Supporting Information. All tests were two‐sided with α = 0.05. Analyses were performed in Stata 18 (StataCorp, College Station, TX).

## 3 Results

### 3.1 Participant Characteristics

**FIGURE 1:**
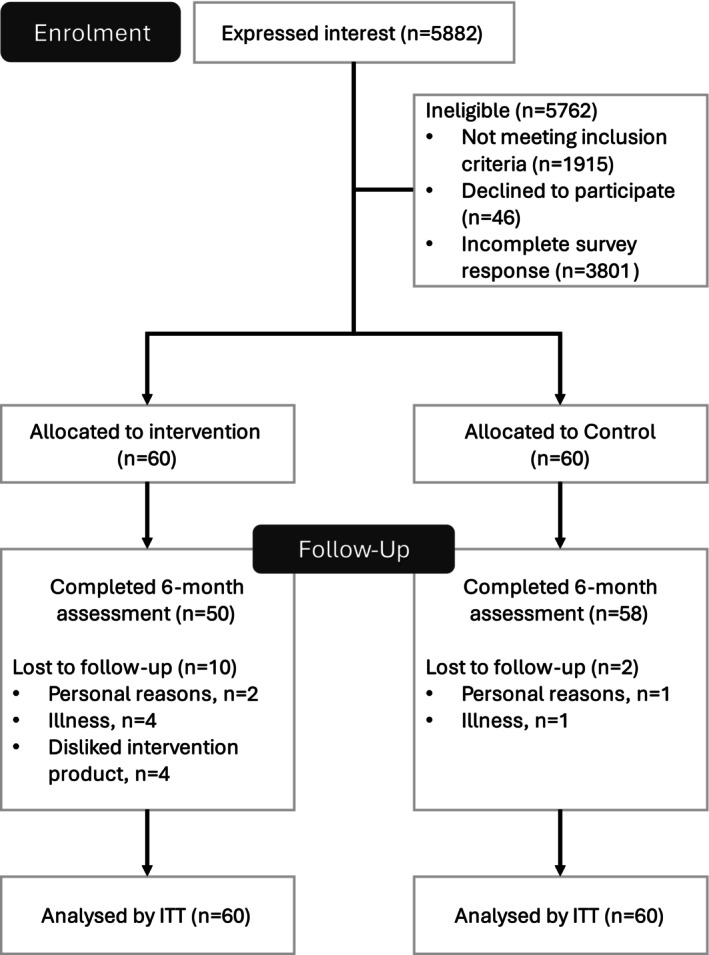
Participant flow chart (analysed by ITT *n* = 60 was based on multiple imputation). *Other reasons—incomplete survey responses.

Overall, 108 (90%) of the 120 participants completed the 6‐month trial (peanut butter group *n* = 50 (83.3%); control group *n* = 58 (96.7%)) (Figure 1). Based on a priori–specified criteria [21], 13 participants in the peanut butter group did not achieve ≥ 80% intervention adherence and were excluded from the per‐protocol analysis. In the peanut butter group, among those who completed the follow‐up, the mean (SD) adherence was 86.0 (13.8) %.

The baseline characteristics of the 120 participants are summarised in Table 1. Overall, 84 participants (*n* = 70%) were females, and the mean ± SD for age and BMI were 76.1 ± 4.6 years and 27.5 ± 4.2 kg/m^2^, respectively, with 40.8% and 30.8% classified as overweight and obese, respectively. In addition, 73.3% (*n* = 44) of participants in the peanut butter group and 85.0% (*n* = 51) in the control group were classified as at risk of sarcopenia (SARC‐F score ≥ 4).

**TABLE 1:** Baseline characteristics by treatment group.

	Peanut butter (*n* = 60)	Control (*n* = 60)
Age, years	75.8 (4.8)	76.4 (4.5)
Sex (female *n*, %)	41 (68.3%)	43 (71.7%)
Current smoker, *n* (%)	2 (3.5%)	2 (3.5%)
Secondary education (Year 9 and below)	2 (3.5%)	4 (6.9)
Anthropometry		
Weight (kg)	76.4 (16.0)	73.3 (13.9)
BMI (kg/m^2^)	27.8 (4.4)	27.2 (4.1)
Overweight | obese, *n* (%)	25 (41.7%) | 19 (31.7%)	24 (40.0%) | 18 (30.0%)
Waist circumference (cm)		
Female	90.9 (12.9)	89.7 (11.5)
Male	102.5 (9.2)	98.3 (11.5)
Medical history, *n* (%)		
Hypertension	31 (51.7%)	34 (56.7%)
Diabetes	3 (5.0%)	4 (6.7%)
Osteoporosis	9 (15.0%)	11 (18.3%)
Heart disease	9 (15.0%)	6 (10.0%)
Habitual physical activity (PASE score)	172.9 (77.5)	187.5 (91.9)
At‐risk of sarcopenia (SARC‐F ≥ 4), *n* (%)	44 (73.3%)	51 (85.0%)
At‐risk of malnutrition (MNA < 24), *n* (%)	5 (8.3%)	3 (5.0%)
HEI‐2020 score	66.5 (9.0)	68.1 (10.1)

*Note:* Values are *n* (%) or mean and (standard deviation), estimated using descriptive statistics (continuous variables) or chi‐square test (categorical variables).

### 3.2 Physical Function, Strength and Muscle Mass

After 6 months, no significant within‐group changes or between‐group differences were observed in the primary outcome of gait speed (Table 2). However, the peanut butter group demonstrated a significant reduction in the time taken to complete 5STS compared to controls (estimated treatment effect, 1.23 s [95% CI, −2.09, −0.37], *p* = 0.006). Similar significant benefits in favour of the peanut group were observed for absolute STS muscle power (estimated treatment effect, 22.0 W [95% CI, 7.1, 36.9], *p* = 0.004) and relative power (estimated treatment effect, 0.27 W/kg [95% CI, 0.10, 0.45], *p* = 0.002), both of which were driven by improvements in the peanut butter group (Table 2). The 30‐s STS performance increased significantly in both the peanut butter (1.3 [95% CI, 0.5, 2.0]) and control groups (0.8 [95% CI, 0.3, 1.3]), with no significant between‐group difference (Table 2). There were no significant within‐group changes or between‐group differences in 30‐s STS, TUG, FSST, muscle strength (both HGS and KES) or any body composition measure (Table 2). Results were consistent with the primary ITT analysis, except that the between‐group difference in HGS approached statistical significance in favour of the peanut butter group (1.4 kg; 95% CI: −0.1 to 2.9; *p* = 0.055) (Table S1).

**TABLE 2:** Mean within‐group change and between‐group differences for changes in physical function over 6 months between the peanut butter and control groups.

	Baseline	6 months	Within‐group change (95% CI)^a^	Estimated treatment effect (95% CI)^b^	*p* ^c^
Gait speed, m/s					
Peanut butter	1.27 (1.22, 1.32)	1.26 (1.20, 1.32)	−0.01 (−0.06, 0.04)	−0.01 (−0.05, 0.04)	0.912
Control	1.22 (1.17, 1.27)	1.24 (1.20, 1.29)	0.02 (−0.02, 0.06)		
5STS, s					
Peanut butter	13.20 (12.37, 14.02)	11.57 (10.90, 12.29)	−1.63 (−2.33, −0.93)*	−1.23 (−2.09, −0.37)	**0.006**
Control	13.60 (12.71, 14.50)	13.12 (12.26, 13.96)	−0.48 (−1.24, 0.27)		
Absolute STS muscle power					
Peanut butter	198.9 (179.2, 218.6)	224.7 (203.8, 245.5)	25.8 (13.6, 37.9)*	22.0 (7.1, 36.9)	**0.004**
Control	181.9 (165.0, 199.2)	186.5 (170.7, 202.3)	4.6 (−4.1, 13.2)		
Relative STS muscle power					
Peanut butter	2.58 (2.40, 2.77)	2.89 (2.70, 3.09)	0.31 (0.16, 0.47)*	0.27 (0.10, 0.45)	**0.002**
Control	2.47 (2.29, 2.66)	2.53 (2.36, 2.70)	0.06 (−0.06, 0.17)		
30‐s STS					
Peanut butter	12.7 (11.8, 13.5)	13.9 (13.0, 14.9)	1.3 (0.5, 2.0)*	0.5 (−0.3, 1.5)	0.207
Control	12.3 (11.4, 13.3)	13.1 (12.2, 14.1)	0.8 (0.3, 1.3)*		
Timed up and go (s)					
Peanut butter	7.68 (7.30, 8.05)	7.89 (7.40, 8.38)	0.21 (−0.23, 0.67)	−0.07 (−0.52, 0.38)	0.758
Control	7.62 (7.38, 7.85)	7.92 (7.65, 8.19)	0.30 (0.10, 0.52)*		
Four‐square step test (FSST) (s)					
Peanut butter	9.79 (9.34, 10.23)	9.29 (8.59, 9.99)	−0.52 (−1.18, 0.13)	−0.12 (−0.85, 0.62)	0.755
Control	10.34 (9.64, 11.03)	9.71 (9.22, 10.19)	−0.64 (−1.14, −0.14) *		
Body weight (kg)					
Peanut butter	76.41 (72.27, 80.55)	76.58 (72.66, 80.50)	0.12 (−0.8, 1.1)	0.20 (−0.82, 1.29)	0.657
Control	73.27 (69.66, 76.89)	73.42 (69.82, 77.02)	0.14 (−0.5, 0.8)		
BMI (kg/m^2^)					
Peanut butter	27.8 (26.7, 28.9)	28.1 (26.9, 29.2)	0.3 (0.0, 0.5)*	0.1 (−0.2, 0.5)	0.571
Control	27.2 (26.2, 28.3)	27.4 (26.4, 28.5)	0.2 (−0.0, 0.4)		
Total fat mass (kg)					
Peanut butter	28.4 (25.9, 31.0)	29.4 (26.8, 32.0)	0.9 (−0.5, 2.4)	0.6 (−0.9, 2.2)	0.433
Control	27.3 (25.1, 29.5)	27.6 (25.4, 29.9)	0.3 (−0.2, 0.8)		
Total lean mass (kg)					
Peanut butter	44.4 (42.1, 46.8)	44.3 (42.1, 46.65)	−0.1 (−0.5, 0.3)	0.2 (−0.3, 0.8)	0.331
Control	42.5 (40.4, 44.5)	42.2 (40.2, 44.3)	−0.3 (−0.54, 0.11)		
Appendicular lean mass (kg)					
Peanut butter	19.9 (18.7, 21.1)	20.0 (18.8, 21.1)	0.1 (−0.2, 0.3)	0.1 (−0.2, 0.4)	0.365
Control	18.9 (17.8, 20.0)	18.9 (17.8, 20.0)	0.0 (−0.2, 0.2)		
Hand grip strength (kg)					
Peanut butter	28.7 (26.5, 30.1)	29.3 (27.2, 31.4)	0.6 (−0.4, 1.7)	0.9 (−0.3, 2.3)	0.148
Control	26.4 (24.6, 28.1)	26.5 (24.8, 28.2)	0.1 (−0.8, 1.0)		
Isometric knee extensor strength (kg)					
Peanut butter	23.6 (19.7, 27.5)	21.5 (19.0, 24.1)	−2.1 (−5.6, 1.6)	0.6 (−2.0, 3.2)	0.629
Control	21.0 (19.0, 22.9)	20.1 (18.0, 22.2)	−0.9 (−2.2, 0.5)		
Disability limitation					
Peanut butter	73.5 (70.5, 76.6)	80.8 (77.3, 84.3)	7.3 (4.6, 10.0)*	0.7 (−2.6, 3.9)	0.673
Control	75.3 (72.0, 78.7)	81.7 (78.0, 85.3)	6.3 (4.4, 8.2)*		
Disability frequency					
Peanut butter	54.7 (51.8, 57.7)	56.0 (52.9, 58.9)	1.3 (−2.1, 4.6)	1.2 (−1.9, 4.3)	0.447
Control	55.3 (52.2, 58.4)	55.5 (52.8, 58.2)	0.2 (−1.6, 1.9)		

*Note:* All analyses were based on 20 imputed data sets.

^a^ Within‐group change (95% CI) was estimated using linear regression models with change scores as the outcome.

^b^ Estimated treatment effects were analysed using linear regression models adjusted for age, sex, baseline values of outcome, BMI, PASE score and HEI‐2020.

^c^ *p*‐values were obtained from linear regression models adjusted for age, sex, baseline values of the outcome, BMI, PASE score and HEI‐2020.

* *p*‐value < 0.005.

The LLFDI disability component limitation score increased in both the peanut butter group (mean change, 7.3 [95% CI, 4.6, 10.0]) and the control group (6.3 [95% CI, 4.4, 8.2]). However, no significant between‐group differences were observed in the change in LLFDI limitation or frequency scores (Table 2).

### 3.3 Dietary Intake

Changes in energy and nutrient intakes are presented in Table 3. There was a significantly greater (312 kcal/day [95% CI, 139, 486], *p* = 0.001) increase in energy intake in the peanut butter compared to control group, explained by a 256 kcal (95% CI, 99, 414) increase in the peanut butter group, with no significant change in the control group (−17 kcal; 95% CI, −125, 90). Absolute protein and total fat intake increased significantly in the peanut butter group compared to controls, by 10.5 g/day (95% CI, 0.5, 21.0, *p* = 0.040) and 23.7 g/day (95% CI, 14.7, 32.8, *p* < 0.001), respectively. Additionally, there was a significantly greater reduction in the percentage of energy derived from carbohydrates in the peanut butter group compared to the control group (estimated treatment effect, −3.7% [95% CI, −6.0 to −1.3], *p* = 0.003). This reduction was due to a significant decrease of −3.1% (95% CI, −4.7, −1.4) in the peanut butter group, while the control group showed no significant change (−0.2% [95% CI, −2.4, 1.9]).

**TABLE 3:** Mean within‐ and between‐group differences in energy/nutrient intakes over 6 months between the peanut butter and control groups.

	Baseline	6 months	Within‐group change (95% CI)^a^	Estimated treatment effect (95% CI)^b^	*p* ^c^
Energy (kcal)					
Peanut butter	1862 (1732, 1992)	2118 (1967, 2270)	256 (99, 414)*	312 (139, 486)	**0.001**
Control	1759 (1613, 1904)	1742 (1598, 1885)	−17 (−125, 90)		
Carbohydrate (g)					
Peanut butter	187.5 (168.3, 206.7)	192.8 (175.2, 210.4)	5.3 (−11.7, 22.2)	13.7 (−3.5, 31.0)	0.116
Control	186.9 (166.8, 207.0)	178.8 (163.2, 194.5)	−8.1 (−20.0, 3.9)		
Carbohydrate (%TE)					
Peanut butter	37.8 (35.6, 40.1)	34.7 (32.7, 36.7)	−3.1 (−4.7, −1.4)*	−3.7 (−6.0, −1.3)	**0.003**
Control	40.1 (37.7, 42.4)	40.1 (38.0, 42.4)	−0.2 (−2.4, 1.9)		
Protein (g)					
Peanut butter	82.1 (76.7, 87.6)	95.5 (87.8, 103.2)	13.4 (6.3, 20.5)*	10.5 (0.5, 21.0)	**0.040**
Control	80.6 (73.3, 87.9)	84.1 (74.9, 93.4)	3.5 (−4.4, 11.5)		
Protein (% TE)					
Peanut butter	18.4 (17.3, 19.6)	18.5 (17.5, 19.4)	0.1 (−0.9, 1.1)	−0.6 (−2.0, 0.8)	0.391
Control	18.9 (17.9, 19.9)	19.4 (18.3, 20.5)	0.5 (−0.7, 1.8)		
Protein (g/kg)					
Peanut butter	1.13 (1.03, 1.24)	1.29 (1.18, 1.41)	0.16 (0.06, 0.25)*	0.13 (−0.01, 0.28)	0.064
Control	1.12 (1.03, 1.22)	1.16 (1.05, 1.28)	0.05 (−0.06, 0.16)		
Total fat (g)					
Peanut butter	73.6 (67.8, 79.5)	95.1 (86.9, 103.3)	21.4 (13.8, 29.1)*	23.7 (14.7, 32.8)	**< 0.001**
Control	68.3 (61.8, 74.7)	67.9 (61.1, 74.7)	−0.4 (−6.2, 5.4)		
Total fat (% TE)					
Peanut butter	35.2 (33.5, 36.8)	39.9 (38.2, 41.7)	4.8 (3.1, 6.5)*	5.5 (3.5, 7.6)	**< 0.001**
Control	34.4 (32.6, 36.2)	33.1 (32.2, 35.9)	−0.3 (−2.2, 1.6)		
Dietary fibre (g)					
Peanut butter	23.2 (21.2, 25.1)	26.2 (24.0, 28.4)	3.0 (0.4, 5.6)*	2.2 (−0.7, 5.0)	0.137
Control	23.5 (21.1, 25.8)	24.4 (22.3, 26.5)	0.9 (−1.2, 3.0)		
HEI‐2020					
Peanut butter	66.5 (64.1, 69.0)	71.2 (68.4, 73.9)	4.6 (2.2, 7.1)*	2.3 (−0.8, 5.4)	0.148
Control	68.2 (65.5, 70.9)	70.1 (67.4, 72.7)	1.9 (−0.5, 4.3)		

*Note:* All analyses were based on 20 imputed data sets.

^a^ Within‐group change (95% CI) was estimated using linear regression models with change scores as the outcome.

^b^ Estimated treatment effects were analysed using linear regression models adjusted for age, sex, baseline values of outcome, BMI, PASE score and HEI‐2020.

^c^ *p*‐values were obtained from linear regression models adjusted for age, sex, baseline values of the outcome, BMI, PASE score and HEI‐2020.

* *p*‐value < 0.005.

Per‐protocol analyses included 107 participants (control: 60; peanut butter: 47) with ≥ 80% adherence to the peanut butter intervention, which are presented in Table S2.

### 3.4 Perception of Intervention

Participants' perceptions of the peanut butter supplement (*n* = 50 completers), including appearance, smell, taste, flavour, texture, aftertaste and overall pleasantness, were high and remained stable throughout the intervention. Ratings for taste, flavour and overall pleasantness ranged from 67 to 69 out of 100, appearance and smell from 62 to 67, texture and aftertaste from 58 to 62 and ease of following the intervention from 76 to 79. In response to the question, ‘How often would you eat the peanut butter?’, the most frequent answer (*n* = 19; 38%) was ‘I like this, and I will eat it now and then’.

### 3.5 Adverse Events

At 6 months, three participants in the intervention group raised concerns: One reported loose stools and bloating, one reported mild burping and one expressed concern about elevated total cholesterol and discontinued the peanut butter prematurely, 6 weeks before the planned end date. The reported symptoms can be multifactorial, and they could not be attributed specifically to peanut butter. No peanut‐related allergic reactions or choking/aspiration events were reported.

## 4 Discussion

In this 6‐month RCT in community‐dwelling older adults, we observed no significant differences between the peanut butter and control group for the primary outcome of gait speed, but there was a greater net improvement in 5STS and STS muscle power for the peanut butter group. However, there was no effect on other measures of physical performance, including the TUG, the FSST and 30‐s STS, body composition (lean soft tissue mass and fat mass), muscle strength or the LLFDI limitation and frequency scores.

Peanut butter improved 5STS performance by 1.23 s, considered a small yet clinically meaningful change. Gonzales‐Bautista et al. demonstrated that longitudinal decline in 5STS performance is associated with a higher incidence of activities of daily living (ADL) disability and suggested that a decline of approximately 1 s (10% per year) can be considered as clinically meaningful [31]. Evidence indicates that muscle power declines earlier and more substantially than muscle strength and muscle mass with ageing [32]. Previous study in an older adult population suggests that an increase in relative muscle power by 0.2 to 0.3 W/kg corresponds with transitions from low to medium and from medium to high 5STS muscle power [33]. Therefore, the 0.25 W/kg (95% CI, 0.08, 0.45) higher in relative 5STS muscle power in the peanut butter group compared with controls observed in our study suggests a shift to a higher muscle power category among the participants in the intervention group. Our findings in muscle power are also clinically important because low relative muscle power is an indicator of mobility limitation, and it has been linked to greater odds of self‐reported walking difficulty [34]. Recent findings further indicate that the dynamic nature of muscle power is more crucial for survival and independence than maximal strength [35]. Therefore, an improvement in muscle power documented in our study is of clinical importance.

It may appear counter‐intuitive why peanut butter improved 5STS power only but not other measures of physical function. One possible explanation is that power is the function of both force (muscle strength) and velocity. In this study, participants were able to complete the 5STS in a shorter time (higher velocity); hence, it explains why improvements in 5STS were seen despite no significant improvement in strength.

The alternative variant of the sit‐to‐stand test, the 30‐s STS test, improved in both groups, although this change did not differ between groups. An explanation for this discrepancy could be that the 30‐s STS primarily evaluates lower‐limb muscular endurance and is influenced by factors such as motivation, balance and fatigue. In contrast, the shorter 5STS is strongly associated with maximal lower‐limb muscle power in older adults [36]. Further research is required to better understand these differential responses and the underlying mechanisms through which nuts may affect specific domains of physical function.

Although our study failed to observe the hypothesised improvement in gait speed after peanut butter supplementation, this null finding was consistent with a previous prospective study involving 3289 individuals aged ≥ 60 years from the Seniors‐ENRICA cohort, which also reported no association between nut consumption and gait speed [19]. The ability to detect changes in gait speed may have been limited by a ceiling effect, as participants in this study were relatively well‐functioning at baseline, with mean gait speeds of ~0.9 m/s, which were above the EWGSOP2 cut‐off of 0.8 m/s for low physical performance [37].

We did not observe any improvement in the muscle strength and muscle mass outcomes. It has been suggested that protein intake exceeding the current recommended dietary allowance (RDA, 0.8 g/kg/day) may serve as a strategy to preserve muscle mass and physical function in older adults [38]. This suggestion is based on the premise that aged muscle requires a greater supply of amino acids to optimally stimulate muscle protein synthesis, particularly in the context of anabolic resistance, which is characterised by a reduced muscle protein synthesis response to anabolic stimuli [39]. Although peanuts are among the nuts with the highest protein content, the amount of peanut butter used in our study (43 g) provided approximately 13 g of protein per day, which may be insufficient to counteract this anabolic resistance. A recent systematic review of RCTs showed that protein supplementation without exercise increased muscle mass by 0.12 kg in community‐dwelling older adults, which is consistent with our study, although not statistically significant. In contrast, a systematic review and network of meta‐analyses of 38 RCTs found that a combined intervention of exercise and protein supplementation is the most effective for improving muscle mass, strength and physical function in healthy older adults [40]. In our study, participants had a baseline protein intake of 1.12 g/kg, which exceeds the recommended intake for this age group [38], potentially limiting the potential for further gains from additional protein intake.

The reported increase in energy (256 kcal), protein (13.4 g) and fat intake (21.4 g) observed in the peanut butter group was consistent with the nutrients provided by the 43‐g peanut butter. Although energy intake increased in the peanut butter group, there was no evidence of weight or fat gain. This finding is consistent with previous studies showing that nut supplementation did not lead to weight gain, and a number of underlying mechanisms have been summarised in a previous systematic review [41]. Potential biological mechanisms include the high unsaturated fatty acid content of nuts, particularly monounsaturated (MUFAs) and polyunsaturated fatty acids (PUFAs), which are more readily oxidised and have greater thermogenic effects [42]. In addition, not all fat (energy) from nuts is absorbed [43]. Diet quality (HEI‐2020) was higher in the peanut butter group, consistent with evidence linking nut intake to improved dietary quality [44]. However, the difference was not statistically significant, likely reflecting ceiling effects given participants' relatively high baseline HEI scores (66–68/100).

The high adherence demonstrates that peanut butter supplementation is a feasible dietary strategy for older adults. This is further supported by the sustained ratings of taste, flavour and overall pleasantness, suggesting good acceptability. Peanut is a nutrient‐dense food, and peanut supplementation in a butter form is a simple, practical, age‐appropriate and inexpensive dietary strategy that can be easily adopted by older adults. Its soft texture also makes it particularly suitable for those with dentition problems, a common concern with ageing [14], and compared to whole peanuts, nearly all its nutrients are available for absorption [14].

Strength of this included a comprehensive assessment of physical function and body composition outcome tests and data on important potential confounders such as diet quality and physical activity levels. We also achieved a high adherence to the peanut butter consumption. This study has limitations. Our sample was generally well‐nourished (112 [93.3%]), with average protein intake exceeding current recommendations, and demonstrated relatively high baseline physical function (higher than EWGSOP cut‐off). In such populations, it is challenging to detect meaningful changes in functional outcomes, as improvements are more likely to be observed in individuals who are malnourished, have inadequate protein intake or have functional impairments, as reported in previous studies [45]. Adherence was evaluated based on the return of unused peanut butter containers. This method, though commonly used, is susceptible to bias (participants may not return unused PB). A more objective method could be used, e.g., the assessment of plasma vitamin E (abundant in peanuts), but this method is expensive and more invasive (requires venepuncture). Participation was voluntary and involved daily peanut butter consumption. It is possible that participants in this study had a more favourable attitude towards peanut butter than the general population and contributed to the high adherence to intervention food in this study. The control group received usual care without a placebo food, and participants were not blinded to allocation, which may have introduced expectancy or behavioural effects. However, all participants were asked to maintain their usual dietary and physical activity habits regardless of group randomisation, and dietary intake and physical activity were measured at the beginning and end of the study and did not change from baseline in both groups. However, all participants were asked to maintain their usual dietary and physical activity habits regardless of group randomisation, and dietary intake and physical activity were measured at the beginning and end of the study and did not change from baseline in both groups. The sample size calculation assumed a relatively large gait speed effect that may have been optimistic for our well‐functioning, well‐nourished participants. No adjustment for multiplicity was applied, increasing the risk of type I error. Instead, we reported effect sizes, 95% confidence intervals and *p*‐values to enable readers to use their own judgement about the relative weight of the conclusions on the effect of the intervention. Although reported energy intake increased in the peanut butter group, we did not observe corresponding changes in body weight or body composition. Since a reduction in energy intake due to dietary compensation was not observed through our dietary measures, lower metabolisable energy from nuts and increased in energy expenditure may be other potential mechanisms explaining these observations. However, since energy expenditure was not measured in this study, we are not able to confirm this speculation and should be explored in future studies. Fall risk was defined using a previously published simplified screening questionnaire, and the predictive validity of this tool for future falls is uncertain. Our findings may not be generalised to population with different fall‐risk profiles or of specific ethnic backgrounds. This study did not assess inflammatory or oxidative stress markers; hence, we were not able to verify the biological pathways that explain how peanut butter might influence muscle power. Future studies incorporating key biomarkers are warranted to better understand the underlying mechanisms.

In conclusion, this 6‐month RCT demonstrated that peanut butter supplementation had no effect on gait speed or other measures of physical function, muscle strength or body composition, but resulted in enhanced 5STS performance and muscle power and high adherence in older adults. Peanut butter was feasible for this population, evidenced by high adherence. Future studies are warranted to investigate the potential benefits of peanut butter supplementation among older adults with low habitual protein intake and low muscle mass or physical function.

## Funding

This work is funded by the Peanut Institute, USA (grant number 980438). The funder had no role in the study design, data collection or analysis, decision to publish or preparation of the manuscript.

## Ethics Statement

The study is approved by the Deakin University Human Research Ethics Committee (2022‐279). Written informed consent was obtained from all participants at the first visit prior to any assessment. The trial was conducted in accordance with the principles of the Declaration of Helsinki and Good Clinical Practice guidelines.

## Conflicts of Interest

The authors declare no conflicts of interest.

## Supporting information


**Table S1:** Mean within‐group change and between‐group differences for changes in physical function over 6 months between the peanut butter and control groups based on per‐protocol analysis.

**Table S2:** Mean within‐group and between‐group differences in energy/nutrient intakes over 6 months between the peanut butter and control groups based on per‐protocol analysis.
