# Supplementary material for: Effects of Peanut Butter Supplementation on Older Adults' Physical Function: A 6‐Month Randomised Controlled Trial
Source: J Cachexia Sarcopenia Muscle. 2026 Feb 3;17(1):e70221. doi: 10.1002/jcsm.70221 (PMC12867520; doi:10.1002/jcsm.70221)
Supplement: Supplementary file 1 — Table S1: Mean within‐group change and between‐group differences for changes in physical function over 6 months between the peanut butter and control groups based on per‐protocol analysis. [file JCSM-17-e70221-s001.docx]

Supplementary Table 1. Mean within-group change and between-group differences for changes in physical function over 6 months between the peanut butter and control groups based on Per-protocol analysis

|  | Baseline | 6-months | Within-Group  Change (95% CI) ^1^ | Estimated treatment effect (95% CI) ^2^ | P-value ^3^ |
| --- | --- | --- | --- | --- | --- |
| Gait Speed, m/sec | | | | | |
| Peanut Butter | 1.27 (1.21, 1.33) | 1.27 (1.20, 1.34) | -0.01 (-0.06, 0.04) | 0.01 (-0.04, 0.06) | 0.716 |
| Control | 1.22 (1.17, 1.27) | 1.24 (1.20, 1.29) | 0.02 (-0.02, 0.06) |  |  |
| 5STS, sec | | | | | |
| Peanut Butter | 13.04 (12.15, 13.94) | 11.43 (10.61, 12.26) | -1.61 (-2.36, -0.87) * | -1.37 (-2.32, -0.42) | 0.006 |
| Control | 13.60 (12.70, 14.51) | 13.10 (12.25, 13.95) | -0.50 (-1.23, 0.24) |  |  |
| Absolute STS muscle power | | | | | |
| Peanut Butter | 203.7 (180.0, 228.0) | 230.8 (206.7, 254.9) | 27.1 (12.2, 41.9) * | 27.2 (12.4, 42.1) | 0.001 |
| Control | 182.2 (165.1, 199.2) | 186.3 (170.4, 202.1) | 4.1 (-4.5, 12.7) |  |  |
| Relative STS muscle power | | | | | |
| Peanut Butter | 2.62 (2.40, 2.84) | 2.96 (2.73, 3.20) | 0.34 (0.15, 0.53) * | 0.32 (0.14, 0.49) | 0.001 |
| Control | 2.47 (2.29, 2.66) | 2.53 (2.36, 2.70) | 0.06 (-0.06, 0.17) |  |  |
| 30 s STS | | | | | |
| Peanut Butter | 12.9 (11.8, 13.8) | 14.1 (12.9, 15.2) | 1.2 (0.3, 2.1) * | 0.7 (-0.3, 1.7) | 0.159 |
| Control | 12.3 (11.4, 13.3) | 13.1 (12.2, 14.1) | 0.8 (0.3, 1.3) * |  |  |
| Timed-up-and-go (s) | | | | | |
| Peanut Butter | 7.64 (7.20, 8.08) | 7.91 (7.29, 8.52) | 0.27 (-0.30 0.83) | -0.03 (-0.57, 0.50) | 0.900 |
| Control | 7.62 (7.38, 7.85) | 7.92 (7.66, 8.20) | 0.30 (0.10, 0.52) * |  |  |
| Four-square step test (FSST) (s) ^1^ | | | | | |
| Peanut Butter | 9.81 (9.28, 10.34) | 9.27 (8.33, 10.22) | -0.54 (-1.46, 0.39) | -0.12 (-1.02, 0.78) | 0.790 |
| Control | 10.34 (9.64, 11.03) | 9.70 (9.20, 10.18) | -0.64 (-1.14, -0.15) * |  |  |
| Body weight (kg) | | | | | |
| Peanut Butter | 76.51 (72.05, 80.97) | 77.22 (73.07, 81.36) | 0.71 (-0.22, 1.63) | 1.05 (-0.04, 2.13) | 0.058 |
| Control | 73.27 (69.66, 76.89) | 73.43 (69.83, 77.02) | 0.16 (-0.50, 0.81) |  |  |
| BMI (kg/m^2^) | | | | | |
| Peanut Butter | 27.8 (26.7, 28.9) | 28.1 (26.9, 29.2) | 0.3 (0.0, 0.5) * | 0.3 (-0.1, 0.6) | 0.191 |
| Control | 27.2 (26.2, 28.3) | 27.4 (26.4, 28.5) | 0.2 (-0.0, 0.4) |  |  |
| Total fat mass (Kg) | | | | | |
| Peanut Butter | 27.8 (253, 30.2) | 28.7 (25.5, 31.9) | 0.9 (-1.5, 3.4) | 1.2 (-1.4, 3.8) | 0.363 |
| Control | 27.3 (25.1, 29.5) | 27.0 (24.3, 29.5) | -0.3 (-1.5, 0.7) |  |  |
| Total lean mass (Kg) | | | | | |
| Peanut Butter | 45.2 (42.4, 48.0) | 45.1 (42.3, 47.8) | -0.1 (-0.7, 0.4) | 0.3 (-0.3, 0.79 | 0.276 |
| Control | 42.4 (40.4, 44.5) | 42.2 (40.2, 44.3) | -0.2 (-0.54, 0.11) |  |  |
| Appendicular lean mass (Kg) | | | | | |
| Peanut Butter | 20.2 (18.8, 21.7) | 20.3 (18.9, 21.7) | 0.1 (-0.2, 0.3) | 0.2 (-0.1, 0.5) | 0.240 |
| Control | 18.9 (17.8, 20.0) | 18.9 (17.8, 20.0) | 0.0 (-0.2, 0.2) |  |  |
| Hand grip strength (Kg) | | | | | |
| Peanut Butter | 29.0 (26.3, 31.6) | 30.1 (27.6, 32.7) | 1.1 (-0.1, 2.4) | 1.4 (-0.1, 2.9) | 0.055 |
| Control | 26.4 (24.6, 28.1) | 26.5 (24.8, 28.2) | 0.1 (-0.8, 1.0) |  |  |
| Isometric knee extensor strength (Kg) | | | | | |
| Peanut Butter | 24.9 (20.0, 29.7) | 23.3 (20.3, 26.3) | -1.6 (-6.1, 2.7) | 1.8 (-1.0, 4.7) | 0.240 |
| Control | 21.0 (19.0, 22.9) | 20.1 (18.0, 22.2) | -0.9 (-2.2, 0.5) |  |  |
| Disability limitation | | | | | |
| Peanut Butter | 74.2 (70.8, 77.6) | 80.6 (76.7, 84.5) | 6.4 (3.9, 8.9) * | -0.4 (-3.4, 2.7) | 0.803 |
| Control | 75.3 (72.0, 78.7) | 81.6 (78.0, 85.3) | 6.3 (4.4, 8.2) * |  |  |
| Disability frequency | | | | | |
| Peanut Butter | 53.9 (50.3, 57.76) | 55.8 (52.1, 59.5) | 1.9 (-2.5, 6.2) | 1.6 (-2.1, 5.4) | 0.385 |
| Control | 55.3 (52.2, 58.4) | 55.5 (52.8, 58.2) | 0.2 (-1.6, 1.9) |  |  |

^1^Within-group change (95% CI) was estimated using linear regression models with change scores as the outcome.

^2^ Estimated treatment effects were analysed using linear regression models adjusted for age, sex, baseline values of outcome, BMI, PASE score and HEI-2020.

^3^p-values were obtained from linear regression models adjusted for age, sex, baseline values of the outcome, BMI, PASE score, and HEI-2020.

All analyses were based on 20 imputed data sets.

*p-value <0.005.
