# Supplementary material for: Effects of Peanut Butter Supplementation on Older Adults' Physical Function: A 6‐Month Randomised Controlled Trial
Source: J Cachexia Sarcopenia Muscle. 2026 Feb 3;17(1):e70221. doi: 10.1002/jcsm.70221 (PMC12867520; doi:10.1002/jcsm.70221)
Supplement: Supplementary file 2 — Table S2: Mean within‐group and between‐group differences in energy/nutrient intakes over 6 months between the peanut butter and control groups based on per‐protocol analysis. [file JCSM-17-e70221-s002.docx]

Supplementary Table 2. Mean within-group and between-group differences in energy/nutrient intakes over 6 months between the peanut butter and control groups based on per-protocol analysis

|  | Baseline | 6-months | Within-Group  Change (95% CI) ^1^ | Estimated treatment effect (95% CI) ^2^ | P-value ^3^ |
| --- | --- | --- | --- | --- | --- |
| Energy (kcal) | | | | | |
| Peanut Butter | 1913 (1753, 2072) | 2141 (1965, 2316) | 228 (54, 401) * | 293 (112, 475) | 0.002 |
| Control | 1764 (1616, 1912) | 1740 (1597, 1885) | -24 (-128, 81) |  |  |
| Carbohydrate (g) | | | | | |
| Peanut Butter | 187.8 (165.4, 210.1) | 190.1 (170.3, 210.0) | 2.3 (-14.7, 19.4) | 9.5 (-7.9, 26.7) | 0.280 |
| Control | 187.2 (167.6, 207.0) | 179.4 (163.9, 194.9) | -7.8 (-20.0, 4.1) |  |  |
| Carbohydrate (%TE) | | | | | |
| Peanut Butter | 37.1 (34.5, 39.7) | 39.4 (35.4, 43.4) | 2.3 (-4.7, -1.4) | 0.9 (-3.5, 5.4) | 0.677 |
| Control | 40.3 (38.2, 42.4) | 40.0 (37.3, 42.7) | -0.3 (-2.4, 1.9) |  |  |
| Protein (g) | | | | | |
| Peanut Butter | 85.3 (79.0, 91.6) | 98.3 (89.3, 107.2) | 13.0 (4.3, 21.5) * | 10.9 (-0.5, 22.3) | 0.077 |
| Control | 80.2 (73.2, 87.2) | 84.1 (74.9, 93.4) | 3.9 (-4.0, 11.7) |  |  |
| Protein (% TE) | | | | | |
| Peanut Butter | 18.7 (17.3, 20.1) | 19.0 (17.6, 20.3) | 0.3 (-1.0, 1.6) | -0.5 (-2.2, 1.1) | 0.597 |
| Control | 18.9 (17.9, 19.9) | 19.4 (18.3, 20.5) | 0.5 (-0.7, 1.8) |  |  |
| Protein (g/kg) | | | | | |
| Peanut Butter | 1.17 (1.05, 1.29) | 1.32 (1.18, 1.44) | 0.15 (0.02, 0.27) * | 0.12 (-0.03, 0.28) | 0.113 |
| Control | 1.12 (1.03, 1.22) | 1.16 (1.05, 1.28) | 0.04 (-0.06, 0.16) |  |  |
| Total fat (g) | | | | | |
| Peanut Butter | 76.4 (69.4, 83.4) | 96.9 (87.5, 106.3) | 20.5 (11.9, 29.1) * | 23.2 (13.3, 33.1) | <0.001 |
| Control | 68.1 (61.8, 74.7) | 67.9 (61.1, 74.7) | -0.2 (-6.2, 5.6) |  |  |
| Total fat (% TE) | | | | | |
| Peanut Butter | 35.5 (33.5, 37.5) | 40.3 (38.2, 42.4) | 4.8 (2.6, 6.9) * | 5.9 (3.3, 8.4) | <0.001 |
| Control | 34.4 (32.6, 36.2) | 34.1 (32.3, 35.9) | -0.3 (-2.2, 1.6) |  |  |
| Dietary fibre (g) | | | | | |
| Peanut Butter | 23.7 (21.4, 26.2) | 25.4 (23.1, 28.0) | 1.7 (-1.2, 4.6) * | 1.2 (-1.9, 4.2) | 0.449 |
| Control | 23.6 (21.1, 25.8) | 24.5 (22.3, 26.5) | 0.9 (-1.3, 3.1) |  |  |
| HEI-2020 | | | | | |
| Peanut Butter | 66.9 (64.2, 69.6) | 70.7 (67.5, 74.0) | 3.8 (0.9, 6.8) * | 1.6 (-1.8, 5.1) | 0.347 |
| Control | 68.2 (65.5, 70.9) | 70.1 (67.4, 72.7) | 1.9 (-0.5, 4.3) |  |  |

^1^Within-group change (95% CI) was estimated using linear regression models with change scores as the outcome.

^2^ Estimated treatment effects were analysed using linear regression models adjusted for age, sex, baseline values of outcome, BMI, PASE score and HEI-2020.

^3^p-values were obtained from linear regression models adjusted for age, sex, baseline values of the outcome, BMI, PASE score, and HEI-2020.

All analyses were based on 20 imputed data sets.

*p-value <0.005.
